# Supplementary material for: Stochastic evolution in populations of ideas
Source: Sci Rep. 2017 Jan 18;7:40580. doi: 10.1038/srep40580 (PMC5241820; doi:10.1038/srep40580)
Supplement: Supplementary Material [file srep40580-s1.pdf]

Stochastic evolution in populations of ideas
Supplementary Material

Robin Nicole, Peter Sollich, Tobias Galla

A Limits on birth-death description of Sato-Crutchfield learning

The parameters Γ and λ of the stochastic evolution of ideas we have defined need to be chosen so that all transition rates $T_n^{\pm}$ in Eq. (23) are non-negative. Except in the case of pure replicator dynamics ($\lambda = 0$), this gives constraints on the parameters that depend on population size N, though weakly. The reason is the logarithmic term in the fitness (20), which can get as large as $-\lambda \ln(1/N)$.

For fixed Γ the rates will only remain non-negative if $\lambda \leq \lambda_c$. One can compute a lower bound for λ_c. Firstly, all the transition rates will be positive if and only if the constraint

$$\left| \Delta\pi - \lambda \ln \frac{N-n}{n} \right| \leq \frac{1}{\Gamma} \tag{35}$$

is met for all $0 < n < N$. Since the quantity $\Delta\pi = \pi_1 - \pi_2$ varies linearly with n, it is bounded by $\Delta\pi(1)$ and $\Delta\pi(N-1)$. Applying the triangular inequality to (35) gives:

$$\left| \Delta\pi - \lambda \ln \frac{N-n}{n} \right| \leq \max\left(|\Delta\pi(1)|, |\Delta\pi(N-1)| \right) + \lambda \ln(N-1) \tag{36}$$

As a consequence, all transition rates are positive as long as $\max\left(|\Delta\pi(1)|, |\Delta\pi(N-1)| \right) + \lambda \ln(N-1) \leq 1/\Gamma$. This translates into

$$\lambda \leq \frac{1}{\ln(N-1)} \left(\frac{1}{\Gamma} - \max\left(|\Delta\pi(1)|, |\Delta\pi(N-1)| \right) \right) \tag{37}$$

The right-hand side therefore provides a lower bound on the critical value λ_c. This bound is plotted as a function of N in Fig. S1. While the bound goes to zero for $N \to \infty$, the inverse logarithmic dependence means it does so extremely slowly: the restriction on the allowed range of λ is therefore mild even for very large population sizes ($N \sim 10^8$ and beyond).

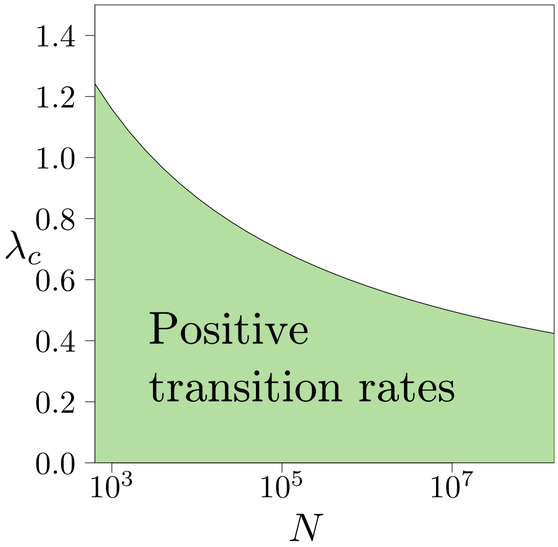

Figure S1. Lower bound on λ_c for a coexistence game as defined in section 3.4, for $\Gamma = 0.1$. The bound (black line) is inversely proportional to the logarithm of the population size N.

B Non-monotonicity of the fixation time in a coordination game

In section 3.4, we studied fixation in a coordination game and observed that the fixation time is *non monotonic* in λ close to the bifurcation threshold λ_c, for *small* N. We will provide an explanation for this phenomenon by decomposing the dynamics leading to fixation into a sequence of elementary events. When N is small enough for activation times to be only moderate, beyond the bifurcation threshold, two additional effects come into play in addition to the relaxation and activation processes observed for large N: (i) direct activation: when starting near $x = 0$, a fluctuation (activation event) can drive the system straight to fixation at $x = 0$, even though the deterministic relaxation would take it in the other direction; (ii) trapping in regions near deterministic fixed points, where the net (deterministic) flow is low; deterministic relaxation times can then become comparable to activation times (precisely at such a fixed point, the deterministic relaxation time is in fact infinite as the flow vanishes). Finite populations will stay trapped in these regions of low deterministic flow for a long (but finite) time. This time will grow logarithmically with N as explained in this Supplementary Material, Sec. D. Such regions exist at and near the bifurcation at λ_c, both for λ below and above λ_c.
The curve in Fig. 6(b) for $\lambda = 0.475$ shows the first effect: for small initial values of x, fixation times are rather low, as direct activation towards $x = 0$ is the dominant fixation mechanism. To the right of the maximum in the curve, on the other hand, we have fixation predominantly at $x = 1$. The fixation time here is, to a good approximation, given by the deterministic relaxation

time to the stable fixed point close to $x = 1$, with the final activation to $x = 1$ being sufficiently fast to be sub-leading.

Accordingly, the sample trajectories in Fig. 7 show that the system moves to the stable fixed point in a close-to-deterministic fashion, with fixation at $x = 1$ occurring shortly afterwards.

The second effect above contributes to the initial condition-dependence of the fixation time in Fig. 6. Here we are close enough to the bifurcation to have an extended region of low flow, causing a significant peak in the transition time curve. The low flow also makes fluctuation effects significant as explained above, and these cause deviations from the times predicted for purely deterministic relaxation. In Fig. 7, the sample trajectories that start from $n = 200$ ($x = 0.2$) illustrate this effect.

Finally, the low flow also makes direct activation to $x = 0$ fast, giving a larger region of initial x where this is the main fixation mechanism. As is clear from Fig. 6(b), the resulting movement of the peak in the fixation time is what causes the non-monotonic λ-dependence at fixed initial condition that is visible in Fig. 6(a). We refer to one of the two sample trajectories starting from $n = 50$ ($x = 0.05$) in Fig. 7 for an illustration of a direct activation event.

We note that the direct activation effects discussed above for the coordination game do occur also for coexistence and dominance games, with the same consequence that fixation times become small for initial conditions near $x = 0$. These other games do not have the additional features arising from the bifurcation in the coordination game, however, so do not show non-monotonic variation of the fixation time with λ.

C Activation dynamics in stochastic evolution of ideas for symmetric games

Here, we explain how to obtain the large N-behaviour of activation times in our stochastic evolution for a population of ideas, and discuss the consequences for the fixation dynamics.

C.1 Kramers-Moyal expansion and effective potential

Our starting point is the dynamics defined by the transition rates (13) and (14). We have discussed in the main text how for $N \to \infty$ this leads to deterministic dynamics, here – by our construction – the Sato-Crutchfield equation (22). This can formally be derived from a Kramer-Moyal expansion to lowest order. In order to capture stochastic effects, one retains the first sub-leading order in the expansion. This is standard for evolutionary processes [1], and

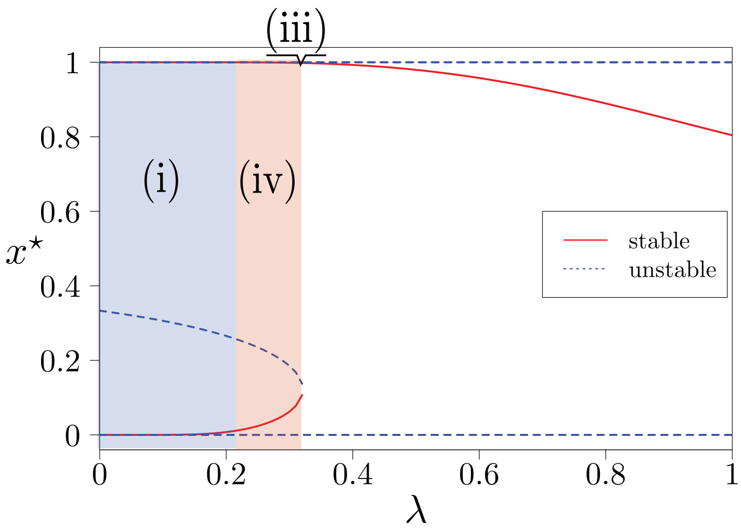

Figure S2. Different types of fixation dynamics in the coordination game with the payoff matrix of Fig. 2, superimposed onto the fixed point structure of Fig. 5(a). For values of λ below the bifurcation threshold, the potential formalism allows one to identify three different zones [(i), (iv) and (iii), with the latter covering only a very narrow λ-range] with qualitatively different fixation dynamics; see Fig. S4. Note that this subdivision into three zones cannot be deduced from the deterministic Sato-Crutchfield dynamics and its fixed point structure (Sec. 3.4) alone.

leads to an Itō stochastic differential equation of the form

$$\dot{x} = h(x) + \frac{1}{\sqrt{N}}\sigma(x)\xi(t), \tag{38}$$

where $\xi(t)$ is Gaussian white noise of unit variance, $\langle \xi(t)\xi(t')\rangle = \delta(t-t')$. For the birth-death process discussed in Sec. 3 one finds

$$h(x) = \Gamma x(1-x)\left(\pi_1(x) - \pi_2(x) - \lambda \ln\left(\frac{x}{1-x}\right)\right), \tag{39}$$

$$\sigma(x) = \sqrt{x(1-x)}. \tag{40}$$

Our aim is to use Eyring-Kramers theory [2], and so we map the above dynamics with multiplicative noise to one with additive noise. This is standard for systems with one degree of freedom, and is achieved by a change of variable from x to

$$y(x) \equiv \int_0^x \frac{\mathrm{d}x'}{\sigma(x')} = 2\arcsin(\sqrt{x}) \tag{41}$$

and conversely $x(y) = \sin^2(y/2)$. Translating the dynamics of x to one for y gives

$$\dot{y}(t) = \frac{h\,(x(y))}{\sigma\,(x(y))} - \underbrace{\frac{1}{2N}\frac{\sigma'(x(y))}{\sigma^2(x(y))}}_{\text{neglected}} + \frac{1}{\sqrt{N}}\xi(t) \tag{42}$$

The additional flow term with prefactor $\frac{1}{N}$ arises from the x-dependence of the original noise variance $\sigma^2(x)$. We will see shortly that this term can be neglected in determining the leading (exponential in N) scaling of activation times. The y-dynamics can now be written in the form

$$\dot{y}(t) = -\Gamma\frac{dV_y}{dy} + \frac{1}{\sqrt{N}}\xi(t) \tag{43}$$

with

$$V_y(y) = -\frac{1}{\Gamma}\int_0^y \mathrm{d}y'\;\frac{h(x(y'))}{\sigma(x(y'))} + \mathcal{O}(1/N) \tag{44}$$

Now that we have a standard Langevin equation with additive noise, Eyring-Kramers theory tells us that the time for an activated event, say from a stable fixed point y_1 to an unstable fixed point (barrier state) y_2 or to a boundary, scales as $\exp\{N\Gamma[V_y(y_2) - V_y(y_1)]\}$. It follows that the $\mathcal{O}(1/N)$ term in V_y will only contribute to the prefactor, which we are not considering here anyway; it can therefore be neglected. More importantly, if we translate back from y to x the potential takes the simple form

$$V(x) = V_y(y(x)) = -\frac{1}{\Gamma}\int_0^x \frac{\mathrm{d}x'}{\sigma(x')}\frac{h(x')}{\sigma(x')} = -\int_0^x \mathrm{d}x'\left[\pi_1(x') - \pi_2(x') - \lambda\ln\left(\frac{x'}{1-x'}\right)\right] \tag{45}$$

and activation times scale as

$$\tau \sim \exp\{N\Gamma[V(x_2) - V(x_1)]\} \tag{46}$$

This will be the basis for our further analysis. In particular, we will exploit that for large N, differences in activation barriers $V(x_2) - V(x_1)$ translate into exponentially different timescales, hence if there are competing processes the one with the smaller activation barrier occurs first (with probability one as $N \to \infty$).

We add finally as a note of caution that the above Langevin analysis is valid for small Γ, where the rates for a transition $n \to n+1$ and its reverse are close to each other. Otherwise a more general approach is needed to determine activation timescales [3].

C.2 Generic symmetric two-strategy games

We can write down the potential $V(x)$ quite generically for a symmetric game where there are two actions to choose from. Inserting the explicit form of the payoffs (see Eq. (4) and (5)) into (45), one has, up to an umimportant additive constant,

$$V(x) = \tilde{v}\left(x - \frac{1}{2}\right) + \tilde{w}\left(x - \frac{1}{2}\right)^2 - \lambda s(x) \tag{47}$$

Here the entropy is $s(x) = -x\ln(x) - (1-x)\ln(1-x)$ as before, and we have introduced the abbreviations

$$\tilde{v} = \frac{a_{21} + a_{22} - a_{12} - a_{11}}{2} \tag{48}$$

$$\tilde{w} = \frac{a_{12} + a_{21} - a_{11} - a_{22}}{2} \tag{49}$$

For $\lambda = 0$ it is now easy to see the link to the three categories of symmetric game considered in Sec. 3.4, bearing in mind that all stationary points of $V(x)$ obey $h(x) = 0$, hence are fixed points of the dynamics. For $w > 0$ and $|v| < w$, $V(x)$ has a minimum in the relevant range $0 \leq x \leq 1$, and we have a *coexistence* game. For $w < 0$ and $|v| < |w|$, on the other hand, $V(x)$ has a maximum, corresponding to a *coordination* game. In the remaining cases, where $|v| > |w|$, $V(x)$ is monotonic for $x \in [0, 1]$, so one has a *dominance* game.

To understand the effect of nonzero λ on $V(x)$, note that the function $-\lambda s(x)$ is convex. Hence for a coexistence game $V(x)$ continues to have a single minimum $x^\star$. A fixation trajectory will first relax to this minimum. The barrier to activation towards $x = 0$ is then $V(0) - V(x^\star)$, so fixation will occur there if this is lower than the corresponding barrier $V(1) - V(x^\star)$ for fixation at $x = 1$. In the opposite case, i.e. for $V(0) > V(1)$, fixation will occur at $x = 1$.

For a dominance game, the inclusion of the entropic term in $V(x)$ will create a single minimum $x^\star$ for any $\lambda > 0$, because the derivative $-\lambda s'(x)$ diverges to $\pm\infty$ at the two boundaries $x = 0$ and $x = 1$. The fixation dynamics then follows the same pattern as for a coexistence game.

C.3 Kramer-Moyal expansion for coordination games

The remaining case of coordination games is the most interesting, as the competition between the maximum in $V(x)$ at $\lambda = 0$ and the convex entropic term can create additional minima. We

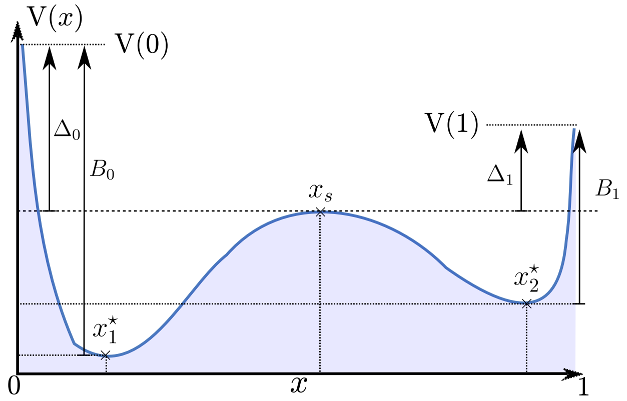

Figure S3. Graphical representation of the definitions of $\Delta_0 = V(0) - V(x_s)$, $\Delta_1 = V(1) - V(x_s)$, $B_0 = V(0) - V(x_1^\star)$ and $B_1 = V(1) - V(x_2^\star)$.

keep $\lambda > 0$ from now on and write

$$V(x) = \lambda \left[v \left(x - \frac{1}{2} \right) + w \left(x - \frac{1}{2} \right)^2 - s(x) \right] \tag{50}$$

with $v = \tilde{v}/\lambda$, $w = \tilde{w}/\lambda$. The shape of $V(x)$ is determined by these parameters, while λ only affects the overall scale of the activation barriers but not their relative size for different processes. We therefore drop the prefactor λ in the following.

For large v and w, corresponding to small λ at fixed $\tilde{v}$ and $\tilde{w}$, the entropic term is mostly negligible in $V(x)$. But its diverging derivative always dominates in $V'(x)$ when one is close enough to the boundaries, so must create two minima there. We denote their positions $x_1^\star$ and $x_2^\star$, respectively, and that of the intermediate maximum by x_s. We also introduce

$$\begin{aligned} \Delta_0 &= V(0) - V(x_s), \qquad & \Delta_1 &= V(1) - V(x_s), \\ B_0 &= V(0) - V(x_1^\star), \qquad & B_1 &= V(1) - V(x_2^\star) \end{aligned} \tag{51}$$

as illustrated in Fig. S3. As v and w change, so will the values of these barrier parameters. In particular, the signs of Δ_0 and Δ_1 determine qualitatively the kind of fixation dynamics that the system will exhibit. The regime where Δ_0 and Δ_1 have *different* signs is subdivided further according to their relation to the barriers B_0 and B_1. A graphical summary is given in Fig. S4 and discussed further below. Fig. S5 shows the resulting phase diagram in the (v, w)-plane, and summarizes to what extent fixation probabilities and fixation times depend on initial conditions in each of the four regimes. Note that when w gets too close to zero, or $|v|/|w|$ becomes too large, a maximum and a minimum of $V(x)$ can merge in a bifurcation. In the single minimum regime beyond this, the fixation dynamics becomes simple again and has the same features as

for coexistence and dominance games. The arrow in Fig. S5 shows how the various regions of the diagram are traversed when λ is increased at fixed $\tilde{v}$ and $\tilde{w}$, i.e. for fixed payoffs. In Fig. S2 we plot over what λ-ranges $V(x)$ has the shapes (i), (iii) and (iv), respectively, in the specific example game of section 3.4. The λ-range for shape (iii) is too small to see in that figure, however.

Fig. S4(a) shows the simplest case $\Delta_0, \Delta_1 < 0$. Here depending on its initial condition, the system will first relax to one of the minima of the potential, say $x_1^\star$. Then because $\Delta_0 < 0$ the barrier for activation to $x = 0$ is smaller than for activation to the maximum x_s. For large N – which we always assume in the following discussion – then with probability one the former process is the first to happen: fixation occurs at $x = 0$. Similarly if the initial relaxation goes to $x_2^\star$ because the system started at $x > x_s$, fixation will occur at $x = 1$. The fixation probability at 0 is therefore a step function of the initial condition x, dropping from one to zero at $x = x_s$. The fixation time changes similarly with initial condition, from $\exp[N\Gamma B_0]$ for $x < x_s$ to $\exp[N\Gamma B_1]$ for $x > x_s$.

The opposite case of $\Delta_0, \Delta_1 > 0$ is illustrated in Fig. S4(b). Here once the system has landed in either of the two minima, it will be able to reach the maximum separating these minima much faster than a boundary. As a result the system will make many "trips" between the two minima and effectively equilibrates across them, forgetting its initial condition. One can show that fixation will then eventually occur as if the system only had a single potential minimum at the *lower* of the two local potential minima, and will accordingly take place at the boundary with the lower value of V.

Finally there is the case where Δ_0 and Δ_1 have opposite signs, e.g. $\Delta_1 > 0$, $\Delta_0 < 0$ as shown in Fig. S4(c,d). If the system starts out of $x < x_s$, we have the same case as (a) above: deterministic relaxation to $x_1^\star$ followed by fixation at $x = 0$ on a timescale set by the barrier B_0. Otherwise, the system will initially relax to $x_2^\star$ and then traverse the maximum at x_s: $\Delta_1 > 0$ ensures that activation to the maximum is exponentially faster than fixation at $x = 1$. After arrival at $x_1^\star$ the earlier sequence of processes is followed. Because fixation in both cases takes place at $x = 0$, the fixation probability is independent of the initial condition.

Whether the fixation time has such a dependence, on the other hand, depends on timescales. As Fig. S4(c,d) shows, the timescale for activation from $x_2^\star$ to x_s is set by the barrier $B_1 - \Delta_1$, while the timescale for fixation at $x = 0$ from $x_1^\star$ is set by B_0. If the former is smaller than the latter, as in Fig. S4(c), then even when the system initially relaxes to $x_2^\star$, the timescale for the overall fixation trajectory will be given by B_0: it is therefore independent of the initial condition. In the converse case of Fig. S4(d), the system will take longer to reach fixation starting from $x > x_s$ because activation from $x_2^\star$ to x_s is much slower than fixation from $x_1^\star$. A typical fixation trajectory here will see the system spend almost all of its time near $x_2^\star$, before a fluctuation

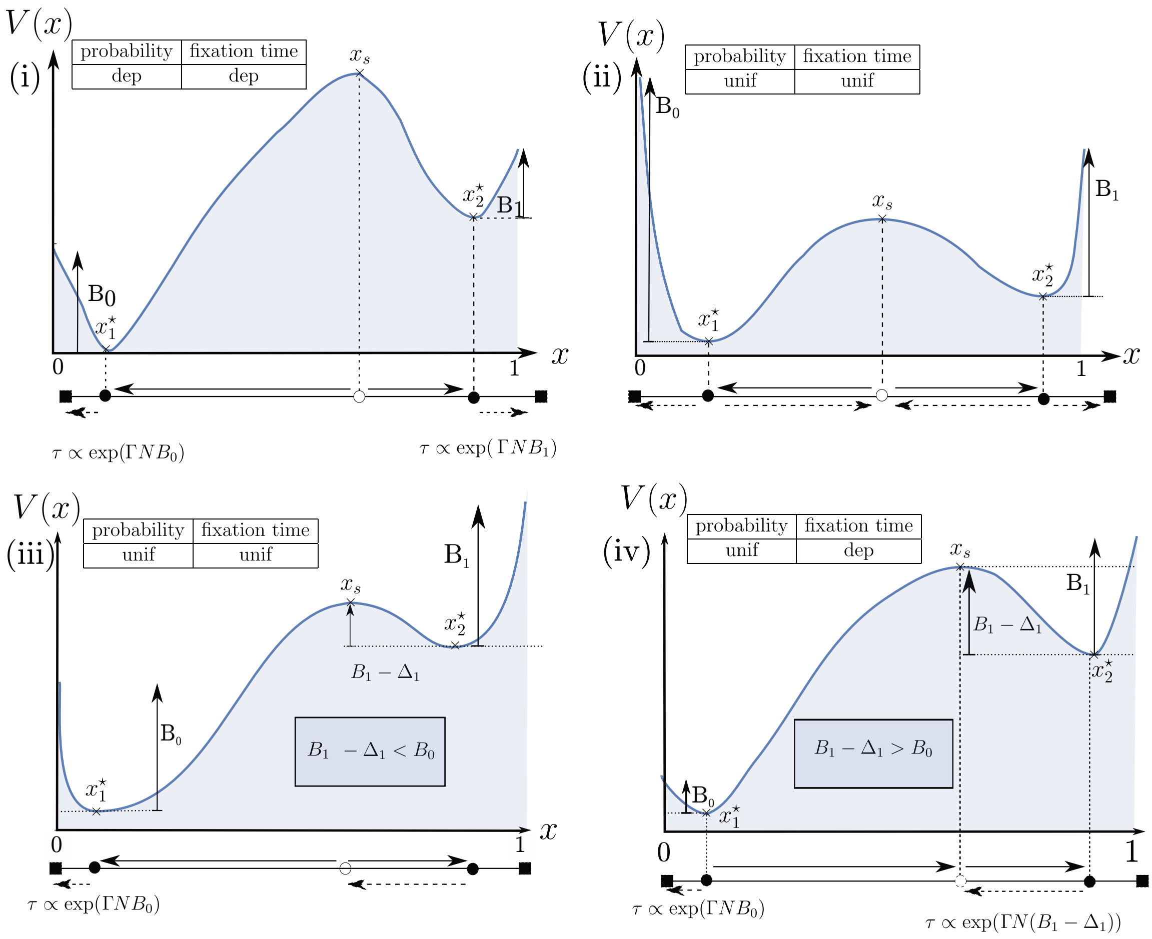

Figure S4. Schematic of the shape of the potential $V(x)$ in the four different classes of coordination games. Arrows on the bottom of each panel represent deterministic relaxation paths that occur during fixation (full lines) as well as activated events driven by fluctuations (dashed lines). The legends indicate whether the large N-fixation probability and fixation time depend on the initial position x, or are uniform in x.
(i) When $\Delta_0 < 0$ and $\Delta_1 < 0$, the barriers to fixation at the boundaries are smaller than the central barrier separating the two potential minima $x_1^\star$ and $x_2^\star$: fixation occurs by deterministic relaxation to one of these points, then activation to the nearest boundary.
(ii) For $\Delta_0 > 0$ and $\Delta_1 > 0$, transitions between the two potential minima are much faster than activated fixation at either boundary. The system equilibrates between the minima, forgetting its initial condition, and fixes at the boundary with lower $V(x)$, here $x = 1$.
(iii,iv) When $\Delta_0 < 0$ and $\Delta_1 > 0$, fixation always occurs at $x = 0$ because from $x_2^\star$ the system will cross the barrier at x_s to $x_1^\star$. **(iii)** If the barrier crossing is faster than the final activation time towards $x = 0$, also the fixation time is independent of the initial condition. **(iv)** Otherwise, the barrier crossing time dominates, causing a much longer fixation time when starting from $x > x_s$.

drives it across x_s to $x_1^\star$ and from there to $x = 0$.

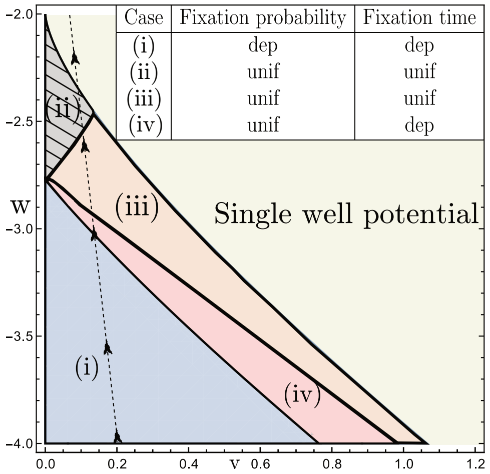

Figure S5. Phase diagram in the (v, w)-plane, indicating where the different shapes of $V(x)$ occur that are explained in Fig. S4. The dotted arrow shows how the phase diagram is traversed at fixed $\tilde{v}$ and $\tilde{w}$ when λ is increased.

D Fixation in regions of small flow

Here, we explain briefly why the noise-driven escape from the low-flow region around an unstable fixed fixed point takes a time scaling as $\ln(N)$.

Consider the linearized dynamics of a coordination (or other) game near an unstable fixed point. After the mapping to Langevin dynamics with additive noise, cf. (43), this can be written in the form

$$\dot{y} = \tilde{\mu}(y - y_0) + \frac{1}{\sqrt{N}}\xi(t) \tag{52}$$

with $\tilde{\mu} > 0$. Assuming that $y(0) = y_0$, a straightforward calculation then shows that the variance of $y(t)$ is:

$$\langle [y(t) - y_0]^2 \rangle = \frac{\exp(2\tilde{\mu}t) - 1}{2\tilde{\mu}N} \tag{53}$$

To have 'escape' from the unstable fixed point this needs to be of order unity; call this value c. Neglecting the -1 in the numerator then gives an escape time of order $t = \ln(2c\tilde{\mu}Nc + 1)/(2\tilde{\mu})$ which for large N becomes $\ln(N)/(2\tilde{\mu})$, establishing the promised logarithmic scaling with N. Note that while we have estimated the time for an escape to a distance of order unity in y-space, this is equivalent to an order unity distance in x-space as the mapping from x to y is smooth.

Bibliography

1. Arne Traulsen and Christoph Hauert. Stochastic evolutionary game dynamics. *Reviews of nonlinear dynamics and complexity*, 2:25–61, 2009.

2. Peter Hänggi, Peter Talkner, and Michal Borkovec. Reaction-rate theory: fifty years after Kramers. *Rev. Mod. Phys.*, 62(2):251, 1990.

3. Peter Hänggi, Hermann Grabert, Peter Talkner, and Harry Thomas. Bistable systems: master equation versus Fokker–Planck modeling. *Phys. Rev. A*, 29(1):371, 1984.
